# New-Onset Diabetes, Endothelial Dysfunction, and Cardiovascular Outcomes in Hypertensive Patients: An Illness-Event Model Analysis

**DOI:** 10.3390/biomedicines9070721

**Published:** 2021-06-23

**Authors:** Raffaele Maio, Edoardo Suraci, Benedetto Caroleo, Cristina Politi, Simona Gigliotti, Angela Sciacqua, Francesco Andreozzi, Francesco Perticone, Maria Perticone

**Affiliations:** 1 Geriatrics Unit, Azienda Ospedaliero Universitaria “Mater Domini”, 88100 Catanzaro, Italy; raf_maio@yahoo.it (R.M.); benedettocaroleo@libero.it (B.C.); 2 Department of Medical and Surgical Sciences, “Magna Graecia” University, 88100 Catanzaro, Italy; edoardosuraci88@gmail.com (E.S.); simona_gigliotti@yahoo.it (S.G.); sciacqua@unicz.it (A.S.); andreozzif@unicz.it (F.A.); perticone@unicz.it (F.P.); 3 CNR-IFC—Institute of Clinical Physiology, 89121 Reggio Calabria, Italy; politicristina89@gmail.com; 4 St. Anna Hospital, 88100 Catanzaro, Italy

**Keywords:** endothelial dysfunction, diabetes mellitus, hypertension, cardiovascular events

## Abstract

Background. Insulin resistance and endothelial dysfunction are common findings in hypertensives, both predisposing to a higher risk of diabetes and cardiovascular events. We designed this study to evaluate the role of endothelial dysfunction in three pathogenetic pathways: (1) from baseline to cardiovascular events, (2) from baseline to diabetes, and (3) from new-onset diabetes to cardiovascular events. Methods. We enrolled 653 Caucasian never-treated hypertensives. Endothelial dysfunction was investigated by strain-gauge plethysmography; incident diabetes and cardiovascular events were evaluated by an illness-event model analysis. Results. During the follow-up (median 113 months), we documented 191 new cardiovascular events and 92 new cases of diabetes. In a multiple Cox regression analysis, acetylcholine-stimulated forearm blood flow [100% decrease, hazard ratio: 2.42 (95% confidence interval = 1.72–3.40)] and serum high-sensitivity C-reactive protein [hazard ratio: 1.30 (95% confidence interval = 1.21–1.40)] had an independent association with cardiovascular outcomes. The incidence rate of cardiovascular outcomes in diabetes-developer patients was higher than in the diabetes-free ones (34.9 vs. 2.5 events per 100 persons-year). In an illness-event model, a 100% decrease in forearm blood flow was associated with a 55.5% hazard ratio increase (hazard ratio: 1.56, 95% confidence interval: 1.33–1.82) of transition 1 (from baseline status to cardiovascular events) and to an almost doubled increase (hazard ratio: 2.54, 95% CI: 2.00–3.25) of the risk of transition 2 (from baseline status to diabetes). No such effects were found in transition 3 (from diabetes to cardiovascular events). Conclusions. Endothelial dysfunction plays a primary role in the pathways leading to diabetes and cardiovascular events in hypertensives. When diabetes is overt, endothelial dysfunction has no predictive value for subsequent cardiovascular events.

## 1. Introduction

Essential hypertension affects over 1 billion people worldwide and is a chronic disorder with a hard global economic and health impact, representing a major risk factor for cardiovascular (CV) disease [1]. In addition, hypertension frequently coexists with insulin resistance, a well-established clinical condition predisposing to a high risk for developing type-2 diabetes mellitus (DM) that, in turn, is both a widespread public health problem, and a risk factor for CV events [2,3,4]. In keeping with this, the coexistence of these two conditions is very frequent also due to the growing prevalence of the same risk factors shared by these two clinical conditions, such as obesity and reduced physical activity, thus CV risk is markedly increased when hypertension and DM coexist [5,6,7].

It is well established that endothelial dysfunction, characterized by a reduced nitric oxide (NO) bioavailability as a consequence of the exposure to cardio-metabolic risk factors, is present in both diabetic and hypertensive patients [8,9]. Interestingly, oxidative stress and the activation of pro-inflammatory pathways represent the most important pathogenetic mechanisms responsible for vascular damage [10,11,12] and the subsequent appearance of fatal and nonfatal CV outcomes [13,14]. On the basis of this evidence endothelial dysfunction can be considered as an intermediate step in the CV continuum from risk factors to clinical events and death.

Even if it is well established that both endothelial dysfunction and DM are associated with an increased risk of fatal and nonfatal CV events [3,4,5,6,13,14], there are no data demonstrating a possible interaction between them. Thus, we designed the present study to assess the effect of endothelial dysfunction, evaluated by strain-gauge plethysmography, in three pathogenetic transitions: (1) from the baseline status to CV events; (2) from the baseline status to DM; (3) from DM to CV events in a population of never-treated hypertensive outpatients free from CV comorbidities and DM at baseline.

## 2. Materials and Methods

### 2.1. Study Population

From a large cohort of 812 newly diagnosed hypertensive subjects participating in the CATanzaro Metabolic Risk factors (CATAMERI) study, we recruited a total of 653 Caucasian patients (337 men and 316 women aged 22–73 years, mean age 48.5 ± 10.5 years), with systolic blood pressure (BP) ≥140 mmHg and/or diastolic ≥90 mmHg from September 1994 to March 2006. From the initial cohort of 812 subjects, 17 patients died, 41 were lost to follow-up, 9 refused to continue the study, and 82 were excluded due to missing high-sensitivity C-reactive protein (hs-CRP) determination. The 653 subjects enrolled for the study were followed up for a median time of 113 months (range 26–206).

Exclusion criteria were previous CV events, DM defined as HbA1c ≥6.5% or fasting plasma glucose ≥126 mg/dL, secondary forms of hypertension, chronic kidney disease (estimated-glomerular filtration rate [e-GFR] < 60 mL/min/1.73 m^2^), liver and peripheral vascular disease, and heart failure (diagnosed according to both clinical and echocardiographic criteria).

Secondary forms of hypertension were excluded by means of a standard clinical protocol, including renal ultrasound studies, computed tomography, renal scan, catecholamine, plasma renin activity, and aldosterone measurements.

The study was approved by the Ethics Committee of the Azienda Ospedaliero-Universitaria Mater Domini of Catanzaro (approval No 2012.63, 17 October 2012). All the participants gave their informed written consent to study participation and all the investigations were made according to the principles of the Declaration of Helsinki.

### 2.2. Laboratory Determinations

At the first eligibility visit, all laboratory measurements were performed after a fasting period of at least 12 h. Plasma glucose was determined by the glucose oxidase method (Glucose Analyzer, Beckman Coulter SpA, Milan, Italy). Triglyceride and cholesterol concentrations were measured by enzymatic methods (Roche Diagnostics GmbH, Mannheim, Germany). Serum creatinine was measured by an automated technique based on the measurement of Jaffe chromogen and by the URICASE/POD method (Boehringer Mannheim, Mannheim, Germany) implemented in an auto-analyzer. Values of e-GFR were calculated by using the equation proposed by CKD-EPI. hs-CRP was measured by a turbidimetric immunoassay (Behring).

Insulin resistance (IR) was estimated by homeostasis model assessment (HOMA-IR) according to the following equation:

HOMA = [insulin (μU/mL) × glucose (mmol/L)]/22.5 [15].

### 2.3. Blood Pressure Measurement

Measurements of clinical BP were obtained in the left arm of seated patients, after 5 min of quiet rest, using a mercury sphygmomanometer, with a minimum of three BP readings on three different occasions at least 2 weeks apart. Systolic (SBP) and diastolic BP (DBP) were recorded at the first appearance and the disappearance of Korotkoff sounds. Baseline BP values were the average of the last two of the three consecutive measurements obtained at intervals of 3 min. Patients with a clinical SBP >140 mmHg and/or DBP >90 mmHg and/or use of antihypertensive drugs were defined as hypertensive.

### 2.4. Vascular Function

Evaluation of vascular function was performed at the first eligibility visit. All studies were conducted by the same experienced investigators (R.M., M.P. and A.S.), at 9:00 a.m. after overnight fasting, with the subjects lying supine in a quiet air-conditioned room (22–24 °C). To test vascular reactivity, we used the protocol previously described by Panza [8] and subsequently employed by our group [12,13,16,17,18]. All patients underwent measurement of forearm blood flow (FBF) and BP during intra-arterial infusion of saline, acetylcholine, and sodium nitroprusside at increasing doses. Measurements of FBF and vascular resistance were repeated every 5 min until stable. Endothelium-dependent and endothelium-independent vasodilations were assessed by a dose–response curve to intra-arterial acetylcholine infusions (7.5, 15, and 30 mg mL^−1^ min^−1^, each for 5 min) and sodium nitroprusside infusions (0.8, 1.6, and 3.2 mg mL^−1^ min^−1^, each for 5 min), respectively. Forearm vascular resistance, expressed in arbitrary units (U), was calculated by dividing mean BP by FBF. For the present study, both maximal responses to acetylcholine and sodium nitroprusside were considered for statistical analysis.

### 2.5. Follow-Up and Incident Cardiovascular Events

All patients underwent periodic control visits at least every six months in the outpatient clinic. To improve long-term follow-up, a questionnaire was also mailed to family physicians, and patients were contacted by phone every 6 months. All clinical events had to be validated by a local Committee on the basis of source data (hospital records, death certificates, or other original documents).

During the follow-up, we assessed the following CV events: fatal and non-fatal myocardial infarction (MI), fatal and non-fatal stroke, transient cerebral ischemic attack (TIA), unstable angina, coronary revascularization procedures (bypass surgery or angioplasty), and symptomatic aortoiliac occlusive disease documented with angiography. Diagnosis of acute MI was based on chest pain history, cardiac enzyme measurement, and new ST elevation in at least two contiguous leads. Unstable angina was defined by typical chest pain associated with ischemic electrocardiographic changes and successively documented by provocative tests (treadmill exercise test or/and stress echocardiography, myocardial scintigraphy, or coronary angiography). TIA was defined by physician diagnosis of any sudden focal neurological deficit that cleared completely in <24 h.

According to the American Diabetes Association diagnostic criteria [19], all patients were nondiabetic at enrollment and did not take any drug known to affect glucose metabolism. New cases of DM were confirmed on the basis of the following criteria: (1) presence of more than one classic symptom of hyperglycemia plus either a fasting plasma glucose ≥126 mg/dL or random plasma glucose ≥200 mg/dL, (2) two or more elevated plasma glucose concentrations (fasting plasma glucose ≥ 126 mg/dL, random plasma glucose ≥ 200 mg/dL, or 2-h plasma glucose ≥ 200 mg/dL during oral glucose tolerance testing), and (3) use of an oral hypoglycemic drug or insulin.

For subjects who experienced one of the events considered for this study, the follow-up ended at the first appearance of the event.

### 2.6. Statistical Analysis

Data are expressed as means ± SD or as percentage frequency, and comparisons between groups were made by one-way ANOVA, Student’s *t*-test, or the X2 test, as appropriate, the Bonferroni post-hoc test for multiple comparisons was further performed. The responses to acetylcholine and sodium nitroprusside were compared by ANOVA for repeated measurements and, when the analysis was significant, Tukey’s test was applied. A value of *p* < 0.05 was considered statistically significant.

The events rate is reported as the number of events per 100 patient-years based on the ratio of the number of events observed to the total number of patient-years of exposure up to the terminating event or censor. For patients without events, the date of censor was that of the last contact with the patient.

The association between endothelial function and incidence risk of CV events was analyzed by univariate and multiple Cox regression analyses. Tested covariates included the maximal vasodilatory response to acetylcholine as well as traditional (age, gender, smoking, fasting glucose, serum cholesterol, SBP, and body mass index (BMI) and emerging (fasting insulin, HOMA index, and hs-CRP) CV risk factors. The multiple Cox regression model was constructed by including all variables that turned out to be associated with the incident risk of CV events (*p* < 0.10) at univariate Cox regression analysis. By this strategy, we constructed a Cox model of adequate statistical power (at least 10 events for each variable into the final model). To assess whether a fixed decrease in FBF (−100%) was associated with heterogeneous effects in three pathogenetic transitions (from the baseline status to CV events [transition 1], from the baseline status to DM [transition 2], and from DM to CV events [transition 3]), an illness-event model including FBF, the interaction term between FBF and the three transitions, as well as all variables which were related to CV outcomes with *p* < 0.10 in the multiple Cox Model 1 was fitted [20]. Data are expressed as hazard ratio (HR), 95% CI, and *p*-value. Data were analyzed by STATA 13 for Windows—StataCorp, Lakeway Drive, College Station, TX, USA.

## 3. Results

### 3.1. Study Population

In Table 1, we reported baseline anthropometric, humoral, and hemodynamic characteristics of the whole study population and of different groups stratified on the basis of the development of CV events and/or DM: patients who did not progress to CV events nor to DM (CV−/DM−), patients who progressed to CV events but not to DM (CV+/DM–), and those who progressed to DM+; among DM+ patients, those who also developed CV events were considered in the DM+/CV+ group. There were no statistically significant differences among groups in gender distribution, BMI, DBP, heart rate, total and LDL-cholesterol, triglyceride, uric acid, and basal FBF. On the contrary, progressors to CV events (CV+/DM−) and diabetes (DM+) were older, and had a higher baseline SBP, glucose, insulin, HOMA index, creatinine, and hs-CRP mean values, while e-GFR and HDL-cholesterol values were lower. Significant differences between DM+ and DM+/CV+ groups were observed only in hs-CRP mean values. As expected, the highest response to acetylcholine stimulated FBF was significantly lower in both progressors to CV events (CV+/DM–) and to diabetes (DM+) compared with that in the CV–/DM– group (215 + 90 and 161 + 116 vs. 350 ± 187%; *p* < 0.0001); in contrast, no significant differences were observed in maximal vasodilation induced by sodium nitroprusside infusion (314 ± 103 and 306 ± 118 vs. 319 ± 108%; *p* = 0.562). No significant differences were observed in maximal endothelial-dependent and endothelial-independent FBF increase in DM+ and DM+/CV+ groups.

At the first eligibility visit, none of the patients had been treated with antihypertensive or antidiabetic drugs. In the whole study population, baseline BP values were 149.3/91.1 ± 17.0/11.5 mmHg, with a significant increase in systolic BP in both the CV+/DM– and DM+ groups (152.6 ± 18.1 and 152.0 ± 17.5 mmHg, respectively) in comparison with the CV–/DM– group (147.9 ± 16.4 mmHg). All patients were treated to reduce clinical BP < 140/90 mmHg using standard lifestyle and pharmacological treatment. Diuretics, β-blockers, ACE-inhibitors, calcium channel blockers, angiotensin II receptor antagonists, and α-blockers were used alone or in various associations without significant differences between the groups. Antihypertensive and antidiabetic drugs used in the study population are reported in Table 1. No significant differences among groups were observed in the percentage of patients reaching the recommended BP target (range 62–65%). Similarly, no differences were observed between DM+ and DM+/CV+ groups with regards to the glycemic target.

### 3.2. Follow-Up and Incident Cardiovascular Events

During the follow-up (median 113 months (range 26–206)), there were 191 new CV morbid events (3.1 events/100 patient-years) at the cardiac (*n* = 134), cerebrovascular (*n* = 50), or peripheral vascular (*n* = 7) level. In particular, there were 51 patients with MI (3 fatal), 53 with unstable angina pectoris, 30 with coronary revascularization procedures, 44 with stroke (5 fatal), 8 with transient cerebral ischemia, and 7 with new-onset peripheral occlusive disease. Indications for the revascularization procedures were put forward by physicians not involved in this study.

Of interest, during the follow-up period, 92 of the 653 initially nondiabetic hypertensive subjects developed new DM; the estimated incidence of new diabetes was 1.6 events/100 patient-years. Among patients who developed DM, 66 also developed CV events; thus, of 191 total CV events, 125 occurred in DM-free patients and 66 in subjects who previously developed DM.

### 3.3. Vascular Function

**Figure 1:**
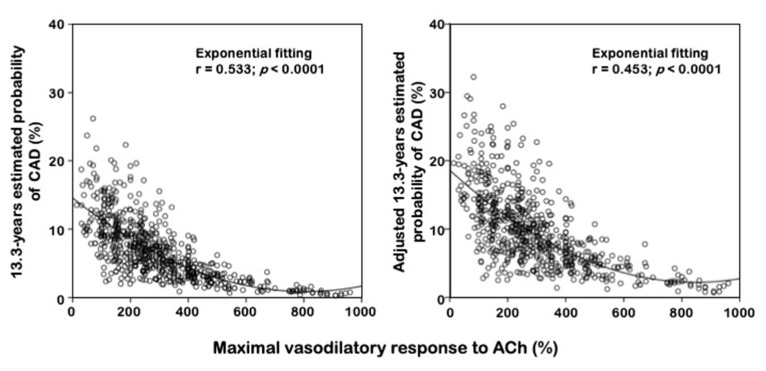
Relationship between endothelium function and risk of cardiovascular events. We graphically reported the relationship, expressed as an exponential fitting curve, crude and adjusted, between maximal vasodilatory response to acetylcholine (Ach) and probability of incident cardiovascular occurrence. Adjusted for age, systolic blood pressure, HDL-cholesterol, HOMA-index, e-GFR, and hs-CRP.

In the whole population, intra-arterial acetylcholine infusion caused a significant dose-dependent increase in FBF and decrease in vascular resistance. The FBF increments from basal (3.35 ± 0.66 mL·100 mL tissue^−1^·min^−1^) at the three incremental doses were 1.84 ± 1.21 (55%), 5.25 ± 3.3 (157%), and 10.0 + 5.9 mL·100 mL tissue^−1^·min^−1^ (298%). At the highest dose of acetylcholine (30 mg/min), FBF increased to 13.35 + 3.9 mL·100 mL tissue^−1^·min^−1^ and vascular resistance decreased to 10.1 ± 4.7 units. Interestingly, when stratifying the study population on the basis of the development of CV events and/or DM, we observed (Table 1) significantly lower values of maximal acetylcholine-stimulated FBF in both CV+/DM– and DM+ groups (215 ± 90 and 161 ± 116 mL·100 mL tissue^−1^·min^−1^, respectively) compared to the CV–/DM– group (350 ± 187 mL·100 mL tissue^−1^·min^−1^). No differences among groups were found for maximal sodium nitroprusside-stimulated FBF. In Figure 1, we graphically reported the relationship, expressed as exponential fitting curve, crude and adjusted, between maximal vasodilatory response to acetylcholine and probability of incident cardiovascular events occurrence.

Similarly, sodium nitroprusside infusion induced (Table 1) a significant increase in FBF (maximal increment from the basal, +317%) and a decrease in vascular resistance (−72%), without any significant difference among groups. Intra-arterial infusion of vasoactive substances caused no changes in BP or heart rate values.

### 3.4. Cox Regression Analyses

On univariate analysis (Table 2), incident risk of CV events was related with a maximal vasodilatory response to acetylcholine (100% decrease, hazard ratio (HR): 2.42 (95% CI = 1.72–3.40), *p* < 0.0001), e-GFR (10 mL/min/1.73 m^2^ increase, HR = 0.79 (95% CI 0.73–0.86), *p* < 0.0001), HDL-cholesterol (10 mg/dL increase, HR = 0.85 (95% CI 0.76–0.97), *p* = 0.013), hs-CRP (HR = 1.42 (95% CI = 1.33–1.51), *p* < 0.0001), HOMA (HR = 1.22 (95% CI = 1.14–1.31), *p* < 0.0001), age (10 years increase, HR = 1.19 (95% CI = 1.03–1.36), *p* = 0.017), and SBP (10 mmHg increase, HR = 1.12 (95% CI = 1.03–1.22), *p* = 0.010). No association was found between occurrence of CV events and smoking, fasting glucose, gender, LDL cholesterol, and BMI.

In a multiple Cox regression model, including baseline variables reaching the statistical significance at univariate analyses, only acetylcholine-stimulated FBF (100% decrease, HR = 1.48 (95% CI = 1.29–1.71), *p* < 0.0001) and serum hs-CRP (HR = 1.30 (95% CI = 1.21–1.40), *p* < 0.0001) maintained an independent association with CV outcomes (Table 2).

**Figure 2:**
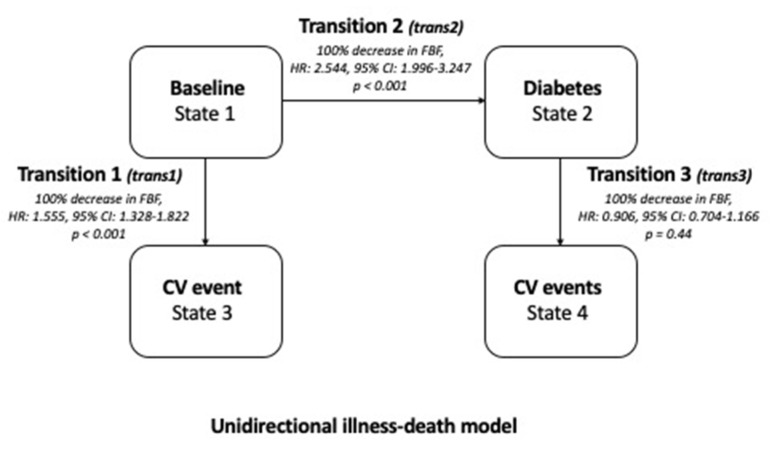
The unidirectional illness-death model. Data are hazard ratios, 95% Cis, and *p*-values. The effect of a fixed decrease (−100%) of FBF was assessed from each transition (see Statistical analysis for more details) according to the endpoint indicated by the arrows.

Across the follow-up period, 92 patients developed de novo type-2 DM. The incidence rate of CV outcomes in patients developing DM (34.9 CV events per 100 persons-year, 95% CI: 27.0–44.4) was significantly higher (*p* < 0.001) than that in DM-free patients (2.50 CV events per 100 persons-year, 95% CI: 2.08–2.97). In an illness-event model, including hs-CRP and e-GFR (see Statistical Analysis section), a 100% decrease in FBF was associated with a 55.5% HR increase in transition 1 (from the baseline status to CV event) and to a more than double increase in transition 2 (from the baseline status to diabetes) (both *p* < 0.001) (Figure 2). Of note, no effect of a 100% decrease in the FBF was found in transition 3 (from DM to CV events) (Figure 2).

## 4. Discussion

In this study, conducted in a very large cohort of never-treated and well-selected hypertensive patients, we demonstrated that endothelial dysfunction and hs-CRP are strong and independent predictors of subsequent CV events and incident DM also after adjustment for some classical and emerging risk factors.

The observed adverse prognostic significance of endothelial dysfunction for future CV events is confirmatory of previously published data [13,14,18] but the novel and clinically relevant, finding obtained in the present study is that the predictive significance of baseline endothelial dysfunction also persists after adjustment for some well-established and strong atherosclerotic vascular risk factors such as age, cholesterol, insulin resistance status, SBP and renal function. Thus, the evidence of a strong association, between baseline endothelial function and subsequent development of CV events in essential hypertension, highlights its relevant pathogenetic role in the appearance and progression of atherosclerotic disease. In this context, since atherosclerosis is characterized by a condition of immune-mediated subclinical inflammation [20,21], it is not surprising that also hs-CRP has emerged as a strong and independent predictor of subsequent CV clinical outcomes. In physiological conditions, the endothelium regulates vascular tone, thrombogenesis, lipid breakdown, vascular inflammation, and smooth muscle cell proliferation. Nevertheless, in the presence of vascular risk factors and/or specific clinical conditions, the endothelium changes its phenotype promoting vasoconstriction, thrombosis, vascular inflammation, and cell proliferation, playing a key pathophysiological role in the development and progression of the atherosclerotic vascular damage [11,22,23] until the appearance of adverse clinical events.

Another relevant clinical finding of this study is that endothelial dysfunction may contribute to the development of type 2 DM, preceding—rather than following—its onset, as a consequence of a low-grade inflammatory state [24] as documented by an hs-CRP increase. Interestingly, this evidence consents to affirm that incident type-2 DM prediction by endothelial dysfunction is able to activate a vicious circle that worsens both vascular damage and glyco-metabolic control with a reciprocal self-empowerment in the induction of CV events. In addition, it is well established that insulin resistance is associated with the activation of the adrenergic system, which reduces vascular reactivity. In keeping with this, we demonstrated in human umbilical vein endothelial cells that angiotensin II, acting via the AT1 receptor, exerts an inhibitory effect on the insulin signaling pathway involved in nitric oxide production, which affects insulin resistance [25].

However, the real novelty of the present study lies in the adverse impact of new-onset DM on CV appearance that, contrary to what is observed in hypertensive patients without DM development, minimizes the pathogenetic role of endothelial dysfunction in the appearance and progression of atherosclerotic vascular damage and subsequent clinical outcomes. It is well demonstrated that type-2 DM is a powerful risk factor for CV events; in fact, individuals with DM have a 2- to 4-fold increased risk of developing vascular outcomes than those without DM [26]. In keeping with this, our hypertensive patients with new-onset type-2 DM showed an incidence rate of CV outcomes higher than those without DM development (34.9 vs. 2.50%). A plausible explanation of this evidence may be recognized in the fact that the coexistence of type-2 DM further impairs the vascular wall dysfunction, already present in hypertensive patients, accelerating the atherosclerotic disease and promoting plaque instability and acute vascular syndromes. In keeping with this, an important adjunctive pathogenetic mechanism may be recognized in the formation of advanced glycation end-products due to chronic hyperglycemia and an excess production of reactive oxygen species (ROS) due to the activation of the polyol pathways. In particular, chronic hyperglycemia impairs cardiac and vascular function through the generation of ROS, which leads to DNA damage and the formation of advanced glycation end-products which, in turn, may have a pivotal role in the development and progression of CV events by inducing vascular fibrosis. In this context, we should not forget the impairment of coronary microcirculation which accentuates the global cardiac ischemic burden of diabetic patients. On the basis of this evidence, it is plausible to affirm that this bidirectional mechanism between endothelial dysfunction and type-2 DM leads to an excess in oxidative stress that determines reduced the NO bioavailability, increased release of inflammatory mediators, and activation of proliferative pathways, all involved in atherosclerosis [11,22]. Finally, present data extend previous knowledge about the complex pathogenetic mechanisms of atherosclerosis, building the hypothesis of a possible hierarchical role of DM upon endothelial dysfunction or, alternatively, an interplay between them on a common pathogenetic pathway leading to atherosclerotic vascular damage and successive clinical CV outcomes. A possible molecular explanation of the predominant role of insulin-resistance upon endothelial dysfunction in the appearance of CV events may be identified in the enhancement of the differentiation of vascular smooth muscle cells to an osteoblast-like phenotype and the consequent increase in vascular stiffness, observed in insulin-resistance status [27]. In this scenario, insulin resistance could be considered as a maker, rather than a marker, of endothelial dysfunction.

## 5. Conclusions

In conclusion, over a very long follow-up period our data demonstrate, that both endothelial dysfunction and new-onset type-2 DM confer an increased risk for CV disease but, notably, with a likely hierarchical role of DM on endothelial dysfunction; in fact, the risk of developing a CV event is higher in subjects with endothelial dysfunction and DM in comparison with those who remain free from type-2 DM. Thus, it is clinically relevant to remark that the occurrence of new-onset type-2 DM is an independent predictor of CV risk in hypertensive patients because these findings emphasize the need to also evaluate insulin resistance status for implementing aggressive strategies to prevent the occurrence of new type-2 DM in hypertensive subjects.

Additionally, even if the evaluation of endothelial function is not a routine procedure, the relevance of our data is that we directly tested endothelial function by stimulating muscarinic cholinergic receptors by intra-arterial infusion of vasoactive agonists. After the beginning of the present study, other methods for the study of endothelial function have been developed and widely applied (i.e., flow-mediated dilation, quantitative angiography), but strain-gauge plethysmography, even if minimally invasive, still remains the gold standard for the evaluation of endothelial function.

Finally, the study has some limitations: it is an observational, non-randomized, prospective study; our findings were obtained in untreated white hypertensives, so results may not be extended to different racial groups or to subjects receiving antihypertensive treatment at the time of the qualifying evaluation.

## Figures and Tables

**Table 1:** Baseline anthropometric, humoral and hemodynamic characteristics of the whole study population and of different groups stratified on the basis of the development of cardiovascular events and/or diabetes.

	All	CV−/DM−	CV+/DM−	DM+	DM+/CV+	*p*
N	653	436	125	92	66	
Gender, *male (%)*	337 (51.6)	228 (52.3)	60 (48)	49 (53.3)	34 (51.5)	0.659 */0.740 ^§^
Age, *years*	48.5 ± 10.5	47.7 ± 10.5	49.4 ± 10.2	50.8 ± 10.6 ^†^	51.4 ± 11.4	0.020 */0.734 ^§^
BMI, *kg/m^2^*	27.5 ± 3.7	27.4 ± 3.7	27.6 + 4.0	27.5 ± 3.1	27.2 ± 3.2	0.936 */0.555 ^§^
Smokers, *n (%)*	107 (16.4)	65 (14.9)	30 (24)	12 (13)	7 (10.6)	0.034 */0.829 ^§^
SBP, *mmHg*	149.3 ± 17.0	147.9 ± 16.4	152.6 ± 18.1 ^#^	152.0 ± 17.5	151.5 ± 16.6	0.006 */0.857 ^§^
DBP, *mmHg*	91.1 ± 11.5	90.6 ± 11.5	92.7 ± 11.5	90.8 ± 11.3	90 ± 11.8	0.188 */0.667 ^§^
Heart rate, *bpm*	72.5 ± 9.7	72.9 ± 9.7	70.9 ± 9.2	72.9 ± 10	71.4 ± 10.2	0.112 */0.358 ^§^
Glucose, *mg/dL*	95.2 ± 10.7	95.0 ± 10.7	94.0 ± 9.5	97.8 ± 11.8 ^‡^	97.5 ± 11.2	0.029 */0.872 ^§^
Insulin, *U/L*	14.4 ± 6.6	13.0 ± 5.4	15.0 ± 5.6 ^#^	20.1 ± 9.6 ^†,‡^	20.1 ± 9.3	0.0001 */1.000 ^§^
HOMA	3.4 ± 1.7	3.1 ± 1.4	3.5 ± 1.4 ^#^	4.9 ± 2.5 ^†,‡^	4.9 ± 2.4	0.0001 */1.000 ^§^
Cholesterol, *mg/dL*	204.6 ± 31.4	204.6 ± 32.3	201.5 ± 28.6	208.6 ± 30.6	209.7 ± 28.4	0.255 */0.819 ^§^
Triglyceride, *mg/dL*	115.5 ± 39.4	113.9 ± 40.0	122.0 ± 40.3	114.3 ± 36.2	108 ± 31.4	00.123 */0.256 ^§^
LDLc, *mg/dL*	129.3 ± 31.4	128.5 ± 12.4	127.8 ± 31.6	135.2 ± 29.2	137.1 ± 28.8	0.011 */0.686 ^§^
HDLc, *mg/dL*	51.6 ± 12.4	52.5 ± 12.4	49.5 ± 11.6	50.4 ± 12.9	50.3 ± 13.2	0.034 */0.962 ^§^
Uric Acid, *mg/dL*	5.1 ± 1.6	5.0 ± 1.6	5.2 ± 1.7	5.0 ± 1.7	5.0 ± 1.8	0.469 */1.000 ^§^
Creatinine, *mg/dL*	0.95 ± 0.2	0.9 ± 0.2	1.1 ± 0.2 ^#^	1.0 ± 0.2 ^†^	1.0 ± 0.2	0.0001 */0.190 ^§^
e-GFR, *mL/min/1.73 m^2^*	85.2 ± 20.0	89.2 ± 19.0	75.4 ± 19.6 ^#^	79.3 ± 19.5 ^†^	75.2 ± 19.9	0.0001 */0.198 ^§^
hs-CRP, *mg/dL*	3.7 ± 1.7	3.2 ± 1.6	4.8 ± 1.5 ^#^	4.5 ± 1.5 ^†^	5.0 ± 1.2	0.0001 */0.026 ^§^
***Forearm Blood Flow,*** ***mL·100 mL tissue^−1^·min^−1^***						
Basal, ACh, *% increase* SNP, *% increase*	3.35 ± 0.66 298 ± 180 317 ± 110	3.40 ± 0.65 350 ± 187 319 ± 108	3.30 ± 0.63 215 ± 90 ^#^ 314 ± 103	3.30 ± 0.70 161 ± 116 ^†^ 306 ± 118	3.30 ± 0.70 154 ± 126 305 ± 117	0.405 */1.000 ^§^ 0.0001 */0.719 ^§^ 0.562 */0.958 ^§^
***Antihypertensive and antidiabetic drugs***						
ACE-i/ARBs, *n (%)*	510 (78)	339 (77.7)	98 (78.4)	72 (78.2)	52 (78.8)	0.985 */0.907 ^§^
Calcium antagonists, *n (%)*	215 (33)	142 (32.6)	42 (33.6)	31 (33.7)	22 (33.3)	0.963 */0.902 ^§^
β-blockers, *n (%)*	59(9)	41 (9.4)	10 (8)	8 (8.7)	6 (9.1)	0.883 */0.843 ^§^
α-blockers, *n (%)*	15 (2)	9 (2.1)	2 (4.8)	4 (4.4)	3 (4.5)	0.350 */0.740 ^§^
Diuretics, *n (%)*	112 (17)	75 (17.2)	21 (16.8)	16 (17.4)	11 (16.7)	0.992 */0.924 ^§^
Associations, *n (%)*	365 (55.9)	243 (55.7)	70 (56)	52 (56.5)	37 (56.1)	0.990 */0.916 ^§^
Anti-diabetics, *n (%)*				75 (81.5)	55 (83.3)	0.934 ^§^
Insulin therapy, *n (%)*				35 (38.1)	25 (37.9)	0.885 ^§^

* *p* < 0.05 by ANOVA among CV−/DM−, CV+/DM−, and DM+ groups, ^§^ *p* < 0.05 by ANOVA between DM+ and DM+/CV+ groups, ^#^ *p* < 0.05 by Bonferroni CV+/DM− vs. CV−/DM−, ^†^ *p* < 0.05 by Bonferroni DM+ vs. CV−/DM−, ^‡^ *p* < 0.05 by Bonferroni vs. CV+/DM−, ACE-i = Angiotensin converting enzyme inhibitors; ACh = acetylcholine; ARBs = Angiotensin II receptor blockers; BMI = body mass index; DBP = diastolic blood pressure; e-GFR = estimated glomerular filtration rate; HDLc = high-density lipoprotein cholesterol; HOMA = homeostasis model assessment; hs-CRP = high-sensitivity C reaction protein; LDLc = low-density lipoprotein cholesterol; SBP = systolic blood pressure; SNP = sodium nitroprusside.

**Table 2:** Cox regression and illness events analyses for incident cardiovascular outcomes.

**Univariate Cox Models Variables**	**Hazard Ratio**	**95% CI**	***p***
FBF, *100% decrease*	2.421	1.724–3.390	0.0001
e-GFR, *10 mL/min/1.73 m^2^*	0.794	0.732–0.862	0.0001
HDL cholesterol, *10 mg/dL*	0.855	0.756–0.967	0.013
hs-CRP, *mg/dL*	1.418	1.330–1.512	0.0001
HOMA	1.218	1.136–1.306	0.0001
Age, *10 years*	1.186	1.031–1.364	0.017
SBP, *10 mmHg*	1.118	1.027–1.216	0.010
Smoking	1.113	0.777–1.593	0.561
Fasting glucose, *10 mg/dL*	1.039	0.907–1.190	0.583
Gender, *male*	1.019	0.766–1355	0.987
LDL cholesterol, *10 mg/dL*	1.009	0.964–1.057	0.691
BMI, *kg/m^2^*	0.997	0.959–1.037	0.889
**Multiple Cox Model 1** **(Including all variables with *p* < 0.10 at univariate Cox analysis)**	**Hazard Ratio**	**95% CI**	***p***
FBF, *100% decrease*	1.484	1.292–1.706	0.0001
hs-CRP, *mg/dL*	1.304	1.214–1.402	0.0001
SBP, *10 mmHg*	1.500	0.964–1.144	0.263
HOMA	1.045	0.963–1.133	0.293
e-GFR, *10 mL/min/1.73 m^2^*	0.922	0.837–1.014	0.095
HDL cholesterol, *10 mg/dL*	0.946	0.838–1.068	0.369
Age, *10 years*	0.998	0.856–1.165	0.984
**Multiple Illness-Event Model** **(Including all variables with *p* < 0.10 at Multiple Cox Model 1)**	**Hazard Ratio**	**95% CI**	***p***
hs-CRP, *mg/dL*	1.248	1.176–1.325	<0.001
e-GFR, *10 mL/min/1.73 m^2^*	0.991	0.984–0.998	0.01
FBF, 100% decrease by Transition 1 interaction term	1.555	1.328–1.822	<0.001
FBF, 100% decrease by Transition 2 interaction term	2.544	1.996–3.247	<0.001
FBF, 100% decrease by Transition 3 interaction term	0.906	0.704–1.166	0.44

BMI = body mass index; e-GFR = estimated glomerular filtration rate; FBF = forearm blood flow; HDL = high-density lipoprotein; HOMA = homeostasis model assessment; hs-CRP = high-sensitivity C reactive protein; LDL = low-density lipoprotein; SBP = systolic blood pressure. Transition 1: from the baseline status to CV event; Transition 2: from baseline status to diabetes occurrence; Transition 3: from diabetes occurrence to CV event (see Figure 2).

## Data Availability

The datasets used and analyzed during the current study are available from the corresponding author on reasonable request.
